# A Novel Signaling Pathway Mediated by the Nuclear Targeting of C-Terminal Fragments of Mammalian Patched 1

**DOI:** 10.1371/journal.pone.0018638

**Published:** 2011-04-13

**Authors:** Hiroki Kagawa, Yuka Shino, Daigo Kobayashi, Syunsuke Demizu, Masumi Shimada, Hiroyoshi Ariga, Hiroyuki Kawahara

**Affiliations:** 1 Department of Molecular Biology, Graduate School of Pharmaceutical Sciences, Hokkaido University, Sapporo, Japan; 2 Department of Biological Sciences, Tokyo Metropolitan University, Tokyo, Japan; Florida International University, United States of America

## Abstract

**Background:**

Patched 1 (Ptc1) is a polytopic receptor protein that is essential for growth and differentiation. Its extracellular domains accept its ligand, Sonic Hedgehog, while the function of its C-terminal intracellular domain is largely obscure.

**Principal Findings:**

In this study, we stably expressed human Ptc1 protein in HeLa cells and found that it is subjected to proteolytic cleavage at the C-terminus, resulting in the generation of soluble C-terminal fragments. These fragments accumulated in the nucleus, while the N-terminal region of Ptc1 remained in the cytoplasmic membrane fractions. Using an anti-Ptc1 C-terminal domain antibody, we provide conclusive evidence that C-terminal fragments of endogenous Ptc1 accumulate in the nucleus of C3H10T1/2 cells. Similar nuclear accumulation of endogenous C-terminal fragments was observed not only in C3H10T1/2 cells but also in mouse embryonic primary cells. Importantly, the C-terminal fragments of Ptc1 modulate transcriptional activity of Gli1.

**Conclusions:**

Although Ptc1 protein was originally thought to be restricted to cell membrane fractions, our findings suggest that its C-terminal fragments can function as an alternative signal transducer that is directly transported to the cell nucleus.

## Introduction

Patched 1 (Ptc1) is a polytopic membrane protein that is an essential component of the receptor for Hedgehog (Hh) signaling [1]–[5]. The Ptc1 signaling pathway regulates a range of processes involved in developmental differentiation, stem cell growth, and cancer etiology [4]–[9]. Malfunction of Ptc1 in mice leads to embryonic lethality, indicating that it is an essential protein in the early development of mammals [10]. In humans, *ptc1* acts as a tumor suppressor gene, as shown by the presence of inactivating mutations in a *ptc1* that occur in sporadic and inherited forms of the common skin tumor, basal cell carcinoma (BCC) [11]–[13], and brain tumors [14], [15]. Thus, it is clear that Ptc1 is crucial for growth and differentiation in vertebrates. Despite its biological importance, the intracellular signaling pathway of mammalian Ptc1 still remains largely elusive.

The downstream pathway of Sonic Hedgehog (Shh) and Ptc1 involves two key proteins, the oncogenic transcription factor Gli and the trans-membrane protein smoothened (Smo) [5], [16], [17]. In the absence of Shh, Ptc1 represses Gli-dependent transcription through Smo inhibition [2], [18]. Extracellular domains of Ptc1 are essential for accepting its ligand, Shh, and binding of Shh alleviates Smo repression, causing activation of Gli1-dependent transcription. In contrast to the understanding of extracellular domain functions, the role of the intracellular domain (ICD) of Ptc1 is rather obscure. Several previous studies imply that the C-terminal end of Ptc1, the seventh and largest intracellular domain (designated Ptc-ICD7 hereafter), is also functional. Ptc-ICD7 was required for appropriate regulation of the Hh signaling pathway in *Drosophila* [19], [20], and deletion of the C-terminal 156 residues of *Drosophila Ptc* was shown to compromise Hh target gene repression [19]. A spontaneous mouse mutant encoding a C-terminally truncated Ptc1 protein [21], [22] showed defects in A-P polarity of the limb bud, and demonstrated increased expression of Shh target genes in white fat tissue [22]. Furthermore, polymorphic variation of Ptc-ICD7 at T1267 was shown to be essential for susceptibility to H-ras-induced squamous carcinoma [23]. In humans, it was revealed that several oncogenic mutations of Ptc1 map to positions corresponding to the ICD7 region [11], [24]–[26]. These findings highlight the functional importance of Ptc-ICD7 in the Hh-mediated signal transduction pathway, though the exact mechanism of Ptc-ICD7 remains unclear.

In *Caenorhabditis elegans*, the transcriptional activity of TRA-1, a single homolog of the mammalian Gli transcriptional factor, is regulated by the *tra-2* gene product [27]–[29] and, indeed, TRA-2 and human Ptc1 share several similar characteristics. They are both twelve-passed transmembrane (TM) proteins (Figure S1A, B), with TM domains arranged in two sets of (1+3+2) membrane-spanning domains reminiscent of some RND-family transporters [17]. TRA-2 is a receptor for secreted glycoprotein HER-1 and modulates the transcriptional activity of TRA-1. The largest seventh intracellular domain of TRA-2 (designated TRA-2 ICD7 hereafter) is located at the C-terminus (Figure S1A, B). Interestingly, several studies indicate that TRA-2 ICD7 has its own biological activity. First, an alternative spliced transcript of *tra-2*, named *tra-2b*, was identified in germline cells of hermaphrodites, and shown to encode a TRA-2 ICD7 fragment [30]. Second, Lum *et al.* (2000) reported that forced expression of exogenous *tra-2b* results in the predominant localization of TRA-2 ICD7 fragment in the nuclei of somatic cells, and causing complete somatic feminization of *XO* animals [31]. This indicates that TRA-2 ICD7 fragment has transcriptional regulatory activity in the nucleus. Third, Kuwabara and co-workers demonstrated that proteolytic cleavage by calpain-like protease TRA-3 generates a soluble ICD7 fragment from the membrane-bound form of TRA-2 [32]. Furthermore, we previously found that the endogenous TRA-2 ICD7 fragment accumulates in the nucleus where it stimulates female-specific transcription in hermaphrodites [33]. These observations indicate that the generation and translocation of the TRA-2 ICD7 fragment into the nucleus is a biologically relevant event in transcriptional regulations in the case of nematodes.

In this study, we found that forced expression of TRA-2 ICD7 fragment in mammalian cells resulted in its clear nuclear accumulation. Stimulated with this observation, we paid our attention to the case in mammalian Ptc1. We found that human Ptc1 protein that was stably expressed in HeLa cells was subjected to proteolytic cleavage at its C-terminus, resulting in the generation of soluble ICD7 fragments. We subsequently observed accumulation of Ptc-ICD7 fragments in the nucleus, while the N-terminal region of Ptc1 remained in the membrane vesicle. Furthermore, we provide conclusive evidence that C-terminal fragments of endogenous Ptc1 accumulate in the nucleus in a wide variety of mammalian cells. Importantly, the ICD7 fragments of human Ptc1 modulate transcriptional activity of Gli1. From these observations, we suggest the existence of a novel alternative signaling pathway for mammalian Ptc1 that is mediated by the generation and nuclear translocation of ICD7 fragments.

## Results

### C-terminal fragments of nematode TRA-2 and human Ptc1 have a potential to localize in the nucleus of human cells

As we reported previously, endogenous fragments of TRA-2 ICD7 accumulate in the nucleus of *C. elegans* hermaphrodite intestinal cells (Figure S2A) [33]. In good agreement with this result, Lum *et al*. reported that GFP-tagged TRA-2 ICD7 fragment, overproduced from the heat shock promoter, localized predominantly to nuclei of nematode somatic cells [31]. In the present study, we show that forced expression of the FLAG-tagged TRA-2 ICD7 fragment in human HeLa cells resulted in clear nuclear localization (Figure S2B). These results potentially indicate that the mechanisms for nuclear translocalization of ICD7 are conserved between nematodes and human.

**Figure 1:**
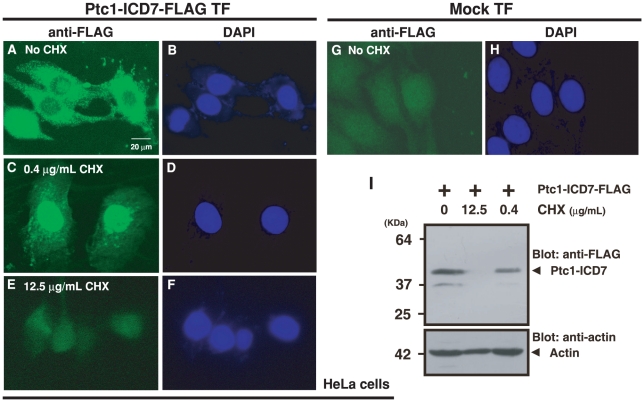
Modest expression of Ptc-ICD7 fragment leads to its nuclear accumulation. C-terminally-FLAG-tagged Ptc-ICD7 cDNA in pCI-neo expression vector was transfected into HeLa cells (Ptc-ICD7-FLAG TF) and immunostained with anti-FLAG antibody. At the time of cDNA transfection, protein synthesis inhibitor cyclohexamide (CHX) was added at indicated concentrations. “No CHX” indicates cell culture without CHX addition (A, B). Cells were harvested 24 hr after transfections. Addition of 12.5 µg/mL CHX completely suppressed protein expression from transfected expression plasmid (E, I), while the addition of 0.4 µg/mL CHX reduced expression of FLAG-tagged Ptc-ICD7 to less than half compared with “No CHX” (I). “Mock-TF” indicates empty vector transfection without CHX addition as a negative control (G, H). Anti-FLAG immunostain in green (A, C, E, G), and DAPI DNA stain in blue (B, D, F, H). Scale bar, 20 µm.

As shown in Figure S1C, ICD7 regions of human Ptc1 and nematode TRA-2 share moderate amino acid sequence similarity (29% identity and 55% similarity). We therefore investigated possible nuclear localization of human Ptc-ICD7 fragments. Forced expression of the C-terminally-FLAG-tagged Ptc-ICD7 fragment in HeLa cells resulted in aggregation in the cytoplasm and the fragment fail to accumulate in the nucleus (Figure 1A, B). However, we found that reduction of the Ptc-ICD7 synthesis rate by the addition of the protein synthesis inhibitor cyclohexamide (at 0.4 µg/mL) clearly induced the nuclear translocation of the Ptc-ICD7 fragment (Figure 1C, D). Immunosignals in the nucleus appear to be specific to the ectopically-expressed FLAG-tagged Ptc-ICD7 fragment, since the addition of higher concentrations of cyclohexamide (12.5 µg/mL, Figure 1E, F) or mock transfection (Figure 1G, H) completely abolished nuclear staining. Western blot quantification indicates that treatment with 0.4 µg/mL cyclohexamide reduced the expression of FLAG-tagged Ptc-ICD7 to less than half of that of “No cyclohexamide” control (Figure 1I). These observations indicate that a moderate rate of Ptc-ICD7 production in cells is essential for its nuclear translocation.

### Stable introduction of full-length *Ptc1* gene into the HeLa cell genome leads to the spontaneous generation of ICD7 fragments

**Figure 2:**
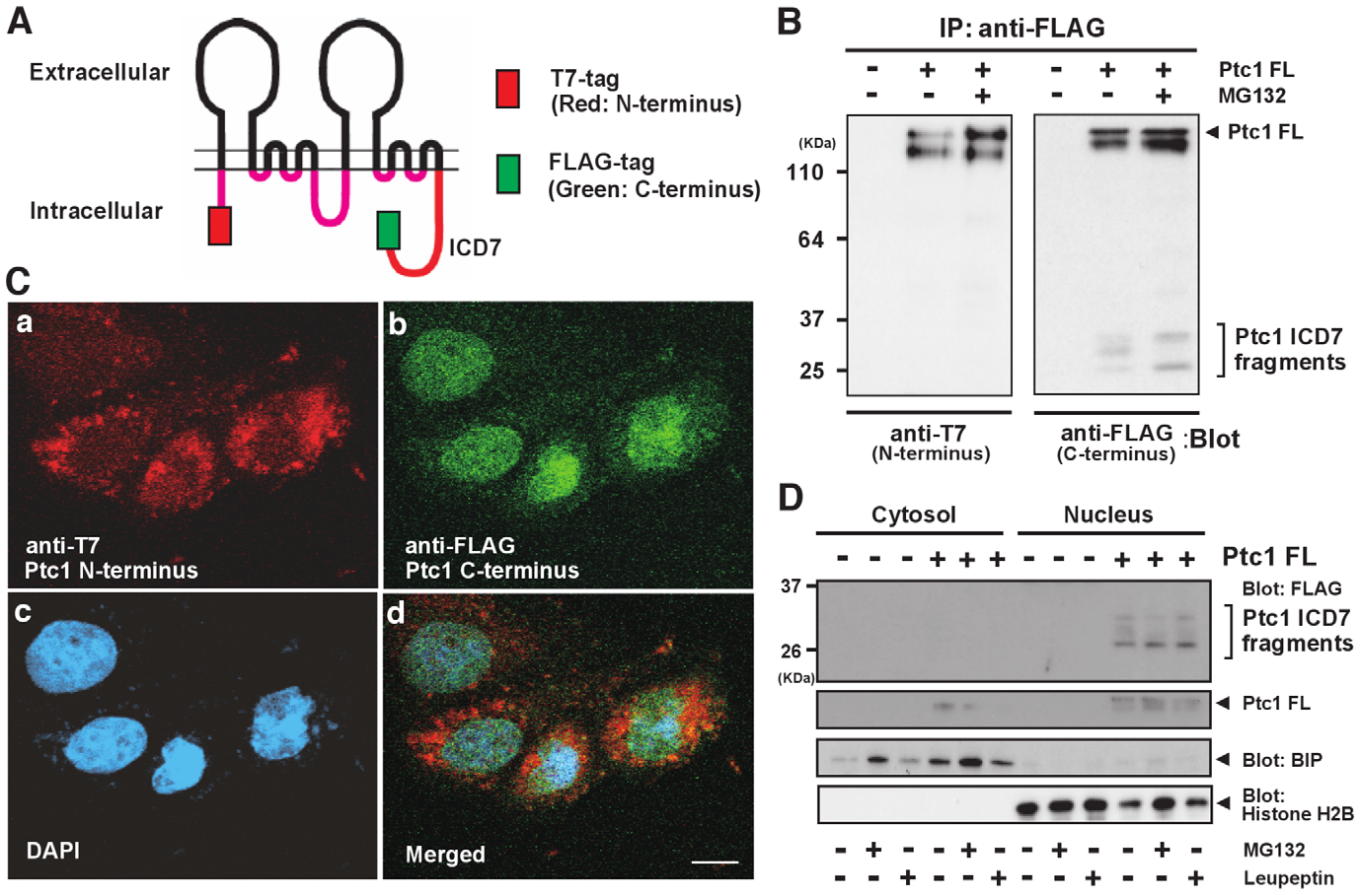
Subcellular localization of stably expressed Ptc1. (A) Schematic representation of stably transfected full-length human Ptc1 protein with possess 12 transmembrane domains. Red box at N-terminus indicates 3×T7-tag, green box at end of intracellular domain indicates C-terminal 3×FLAG-tag, respectively. (B) Western blot analyses of Ptc1 protein stably expressed in HeLa cells. Ptc1 immunoprecipitated from cell extracts with C-terminal FLAG-tag and immunoblotted with anti-N-terminal T7 tag antibody (left panel) or anti-C-terminal FLAG tag antibody (right panel). Anti-T7 blot detects doublet bands corresponding to full-length Ptc1 (Ptc1 FL), while anti-FLAG blot reveals small C-terminal fragments representing the intracellular domain of Ptc1 (ICD7 fragments) as well as Ptc1 FL. (C) Multiple staining of Ptc1-stably expressing cells by anti-T7 tag immunostain (a: Ptc1 N-terminus), anti-FLAG-tag immunostain (b: Ptc1 C-terminus), DAPI DNA stain (c: nucleus) and their merged image (d). Scale bar, 20 µm. (D) Cell fractionation analysis of Ptc1-stably expressing cells. BIP and Histone H2B used as cytoplasmic and nuclear marker, respectively. Ptc1 FL (+), cells stably expressing full-length Ptc1; Ptc1 FL (−), control HeLa cells.

Ptc1 is a twelve-passed trans-membrane protein that is rich in hydrophobic amino acids (Figure S1A, B), so its forced expression in HeLa cells tends to result in cytoplasmic aggregation. To prevent this, we established HeLa cell lines stably transfected with a cDNA fragment encoding human full-length Ptc1 (isoform L). As shown in Figure S3, the expression level of ectopic Ptc1 protein in a stable cell line is less than one fifth of that of transient over-expression. In the stably transfected cell line we used, full-length Ptc1 was fused with an N-terminal 3×T7-tag and C-terminal 3×FLAG-tag (Figure 2A), to distinguish the behavior of either half of this trans-membrane protein. We then performed immunoblot experiments to verify its expression. After immunoprecipitation with anti-FLAG antibody, the N-terminal T7-tag blot clearly showed expression of the 140 kDa full-length form of Ptc1 (Figure 2B, left panel). It should be noted, however, that the C-terminal anti-FLAG-tag blot detected several small species of molecular weights 25–36 kDa, which are likely to correspond to fragments encompassing the intracellular domain of Ptc1, in addition to the expected 140 kDa full-length form of the receptor (Figure 2B, right panel). Unexpectedly, we noticed that pretreatment of cells with 10 µM protease inhibitor MG132 modulated the band pattern of smaller species (Figure 2B, right panel). To further investigate the effects of inhibitors, we examined the behavior of the C-terminal band pattern with a series of protease inhibitors. Our analysis showed that neither calpain nor γ-secretase inhibitors had obvious effects on the processing of Ptc-ICD7 (Figure S5). We found that treatment with Lactacystin, as well as MG132, modulated the appearance of smaller species (Figure S5). Since both Lactacystin and MG132 are known as proteasome inhibitors, these observations indicate that proteolytic cleavage of the C-terminal cytoplasmic domain of stably expressed human Ptc1 is modulated by proteasome, either directly or indirectly, resulting in the formation of soluble Ptc-ICD7 fragments. The precise mechanism of proteolytic cleavage control is, however, currently unknown.

### Subcellular localization of stably expressed human Ptc1

To analyze the subcellular localization of stably expressed human Ptc1, we stained stably transfected cells with anti-N-terminal T7 tag and/or anti-C-terminal FLAG-tag antibodies. With the anti-N-terminal T7-tag antibody, the large cytoplasmic punctuates observed (Figure 2C-a). This localization pattern is consistent with the previously reported cellular localization of endogenous Ptc1 [34]–[36] and may correspond to multivesicular bodies and endosomes, likely representing transit through the secretory and/or endocytic pathways.

By contrast, C-terminal staining with the anti-FLAG-tag antibody was mostly in the nucleus (Figure 2C-b, c), suggesting that they can be cleaved off and transported here. To verify this, we analyzed the distribution of Ptc-ICD7 fragments using cell fractionation method. As shown in Figure 2D, full-length Ptc1 was detectable in both the cytoplasmic and nuclear membrane fraction of HeLa cells, supporting previous observations that most full-length Ptc1 is localized on cytoplasmic membrane fractions. In contrast, the 25–36 kDa C-terminal ICD7 fragments were specifically present in the nuclear fraction (Figure 2D, upper panel). As described previously, pretreatment of cells with MG132 also modulated the band pattern of these nuclear fragments (Figure 2D). Both of these biochemical and immunocytochemical observations indicate that C-terminal fragments of Ptc1 (detected by the antibody against C-terminal FLAG tag) are processed by unidentified endoproteinase and the ICD7 fragments are specifically translocated to the nucleus.

### Establishment of a specific antibody for the C-terminus of Ptc1

**Figure 3:**
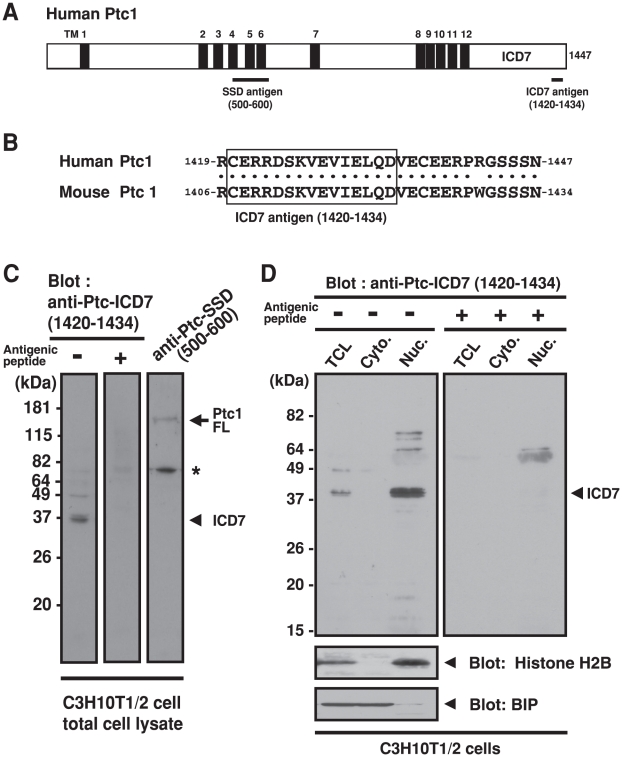
Endogenous Ptc1 ICD7 fragment in C3H10T1/2 cell. (A) Schematic diagram of human Ptc1 protein (isoform L). Twelve transmembrane (TM) domains are indicated by black boxes. Antigenic regions of SSD (500–600) and ICD7 (1420–1434) are indicated by underlines. (B) Amino acid alignment of the C-terminus of Ptc1. Antigenic sequence of anti-Ptc-ICD7 (1420–1434) antibody is indicated by a box. Identical residues in humans and mice are indicated by dots. (C) Western blot analysis with mouse C3H10T1/2 total cell lysate. Endogenous Ptc1 protein in cells was detected with either anti-Ptc-ICD7 (1420–1434) antibody or anti-Ptc SSD (500–600) antibody. Inclusion of competitive antigenic peptide is indicated as (−) or (+). Asterisk indicates unidentified signal. (D) Cell fractionation assay of C3H10T1/2 cells. Each fraction was subjected to immunoblot analysis using anti-Ptc-ICD7 (1420–1434) antibody. TCL: total cell lysate. Cyto.: cytoplasmic fraction. Nuc.: nuclear fraction. Antigenic peptide competition abolished the 37 kDa immunosignal in the nuclear fraction.

The cellular behavior of endogenous Ptc-ICD7 fragments in mammalian cells is enigmatic as an appropriate C-terminal-specific antibody had not been available. We therefore newly established a polyclonal antibody to a synthetic peptide (1420-CERRDSKVEVIELQD-1434) that is derived from the C-terminal sequence of human Ptc-ICD7 (Figure 3A, B). It should be noted that the antigenic sequence is completely conserved between human and mouse (Figure 3B), thus it can be applicable for both of these species. We designate this newly established polyclonal IgG as anti-Ptc-ICD7 (1420–1434) antibody. This antibody recognized both full-length and C-terminal fragments of mammalian Ptc1 that was overexpressed in HeLa cells (Figure S4).

We next examined endogenous Ptc1 protein in C3H10T1/2 cells, mouse embryonic fibroblast cells that have the potential to undergo osteoblast differentiation in response to Shh [37]–[40], indicating that the Ptc1-dependent signaling pathway is active in this cell line. We verified the expression of endogenous Ptc1 protein in this cell line with a commercially available antibody. Western blot analysis of C3H10T1/2 cell lysates with an anti-Ptc1-SSD antibody that recognizes the sterol-sensing domain (SSD, residues 500–600 of human Ptc1, Figure 3A) of mammalian Ptc1 reacted with signal correspond to membrane-assembled form of Ptc1 (Figure 3C, right lane, indicated by an arrow). In identical conditions, we found that our anti-Ptc-ICD7 (1420–1434) antibody detected a 37-kDa signal that might correspond to the endogenous ICD7 fragment of mouse Ptc1 (Figure 3C, left lane, indicated by an arrowhead). This signal was completely absorbed by the addition of excess peptide corresponding to the antigen, indicating that the signal is specific (Figure 3C, central lane). For an unknown reason, we could not detect any endogenous signals that correspond to the full-length form of mouse Ptc1 in C3H10T1/2 cells with anti-Ptc-ICD7 (1420–1434) antibody.

### Endogenous Ptc-ICD7 fragments can be detected in the nucleoplasm of mammalian cells

**Figure 4:**
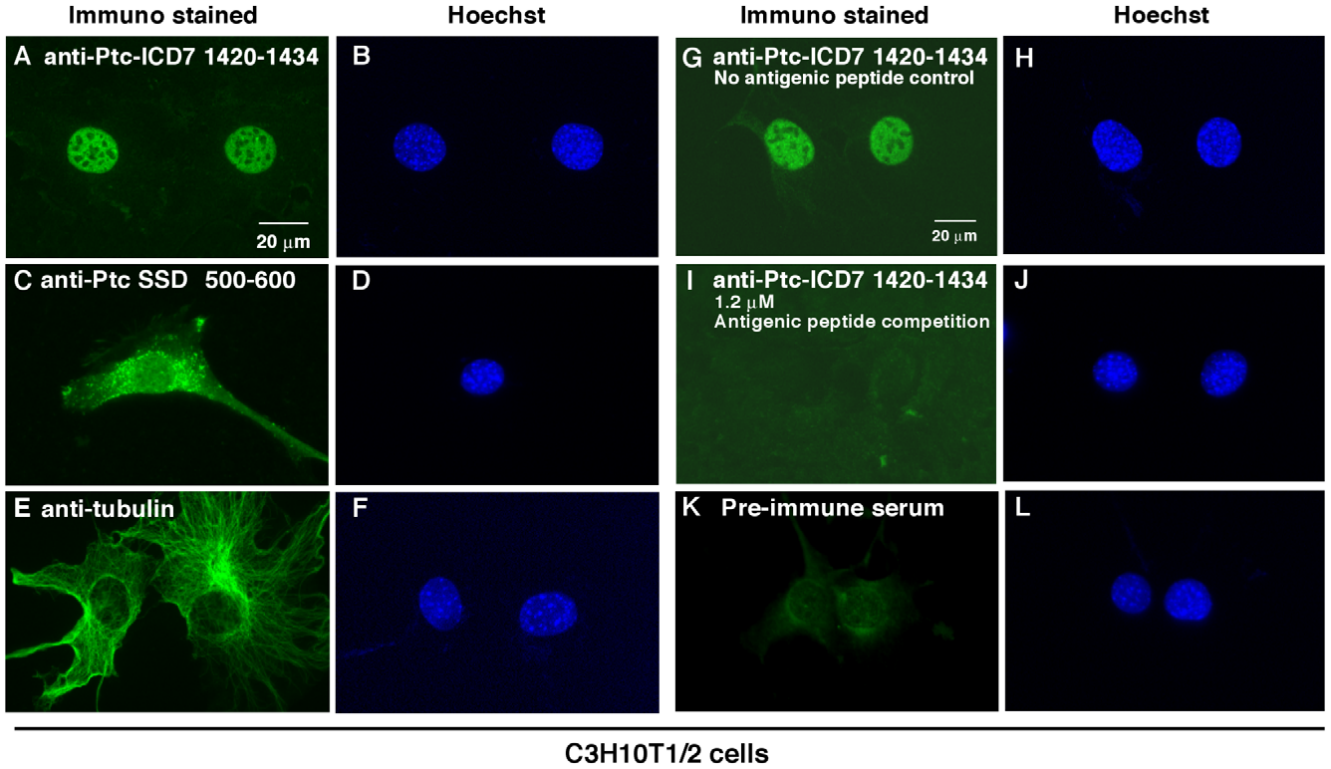
Nuclear localization of endogenous Ptc1 ICD7 fragments in C3H10T1/2 cells. Immunocytochemical analyses of endogenous Ptc1 protein in mouse C3H10T1/2 cells with a series of anti-Ptc1 antibody. Antibodies used are as follows: anti- Ptc-ICD7 (1420–1434) antibody (A), anti-Ptc1 antibody that recognizes SSD (500–600) region (C). Anti-α-tubulin DM1A antibody used as positive control for cytoplasmic immunostaining (E), and “Pre-immune serum” staining used as negative control (K). (G–J) Nuclear immunosignals of endogenous ICD7 fragments were competitively suppressed by corresponding antigenic peptide. 5.5 nM anti-Ptc-ICD7 (1420–1434) primary antibody was absorbed with (I) or without (G) excessive amounts of antigenic peptide. Antigenic peptide concentrations for competition were 1.2 µM. Hoechst DNA stain, blue (B, D, F, H, J, L). Scale bar, 20 µm.

**Figure 5:**
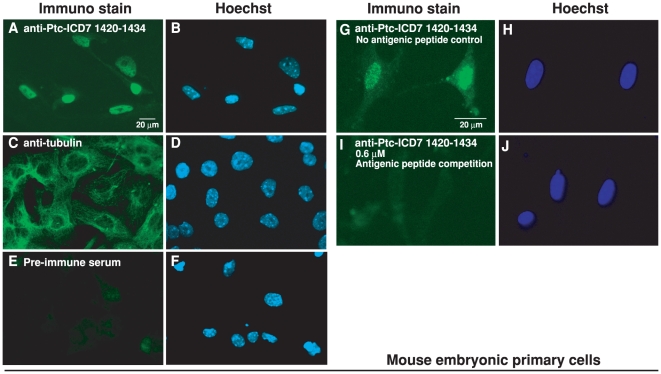
Nuclear accumulation of endogenous Ptc1 ICD7 fragments in mouse embryonic primary cells. Immunocytochemical analyses of endogenous Ptc1 ICD7 fragments in mouse embryonic primary cells. (A) Anti-Ptc-ICD7 (1420–1434) antibody staining. (C) Anti-α-tubulin DM1A antibody as positive control for cytoplasmic immunostaining. (E) Pre-immune serum staining was used as a negative control. 5.5 nM anti-Ptc-ICD7 (1420–1434) primary antibody was absorbed with (I) or without (G) excessive amounts of antigenic peptide. Antigenic peptide concentrations for competition were 0.6 µM. Hoechst DNA stain, blue (B, D, F, H, J). Scale bar, 20 µm.

We next examined the subcellular localization of endogenous Ptc1 protein in C3H10T1/2 cells. Immunocytochemical staining of C3H10T1/2 cells with antibody that specifically recognizes the SSD domain showed punctate cytoplasmic staining that would be expected for intracellular endocytic membrane vesicles (Figure 4C, D). These patterns are consistent with the previously reported cellular localization of full-length Ptc1 [34]–[36]. By contrast, immunosignals of the anti-Ptc-ICD7 (1420–1434) antibody at a final concentration of 5.5 nM showed clear nuclear staining (Figure 4 A, B), suggesting that endogenous fragments of Ptc-ICD7 can be transported into the nucleus. We also provide evidence that C-terminal fragments from endogenous Ptc1 accumulate not only in the nuclei of C3H10T1/2 cultured cell line but also in that of mouse primary cells from 14 dpc embryos (Figure 5A, B). These observations indicating that the nuclear localization of Ptc-ICD7 could be a general event, although strength of nuclear signals was varied from cell to cell in the case of mouse primary cells. These nuclear staining were specific to Ptc1 protein, as immunostaining with non-immune control IgG (Figure 4, K, L, Figure 5, E, F) and anti-α-tubulin antibody (Figure 4, E, F, Figure 5, C, D) showed no nuclear staining under identical experimental conditions as those used in anti-Ptc-ICD7 (1420–1434) antibody staining. To further evaluate antibody specificity for nuclear staining, we performed an antigen competition assay. By absorption using excess amounts of antigenic peptide (at a final concentration of 1.2 µM peptide), we confirmed that immuno-signals of the anti-Ptc-ICD7 (1420–1434) antibody (at a final concentration of 5.5 nM IgG) in the nucleus of C3H10T1/2 cells were completely abolished (Figure 4 I–J). Similarly, nuclear immunosignals in embryonic primary cells were abolished with the 0.6 µM antigenic peptide competition (Figure 5 I–J).

Nuclear-specific accumulation of the endogenous Ptc-ICD7 fragment in C3H10T1/2 cells was further confirmed by a cell fractionation assay. As shown in Figure 3D, C-terminal fragments detected with the anti-Ptc-ICD7 (1420–1434) antibody were specifically present in the nuclear fraction. Again, the immunosignal of 37 kDa in the nuclear fraction is specific since antigenic peptide competition completely abolished the reactivity (Figure 3D). Together, these results indicate that the anti-Ptc-ICD7 (1420–1434) antibody specifically and exclusively recognized corresponding target fragments in the nucleus. We therefore suggest a novel signaling mechanism for mammalian Ptc1 that is mediated by the generation and nuclear translocation of C-terminal ICD7 fragments.

### ICD7 fragments of Ptc1 modulates transcriptional activity of Gli1

**Figure 6:**
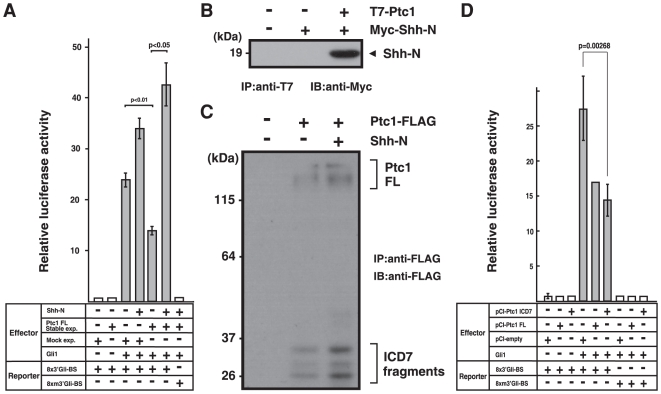
Gli1-based luciferase-reporter assay reveals Shh response in Ptc1 transfected cells. (A) Dual-luciferase reporter assay performed with Gli1 expression vector (Gli1) and a reporter construct consisting of eight copies of the Gli-binding site (8×3′Gli-BS) or its mutated version (8×m3′Gli-BS) as a negative control. Normalization of transfection efficiencies was carried out using *Renilla* luciferase activities as an internal control. Relative luciferase activity was monitored in cells stably expressing full-length Ptc1 (Ptc1 FL Stable exp.) and its negative control (Mock exp.) with (+) or without (−) Shh-N-conditioned media. This medium contains 19 kDa form of Shh-N fragment of Myc-tagged-Shh-N that was processed and secreted from in HeLa cells expressing full-length Shh. Addition of the Shh-N fragment stimulates transcriptional activity of Gli1 in cells stably expressing full-length Ptc1. All reporter assay experiments were repeated at least three times, and transfection was done in duplicate. (B) Stably expressed full-length Ptc1 binds with secreted Shh-N. HeLa cells that were stably expressed N-terminally T7-tagged full-length Ptc1 was exposed to Myc-Shh-N-conditioned medium. After cells were harvested, full-length Ptc1 was immunoprecipitated with N-terminal T7-tag from solubilized cell lysates (IP:anti-T7) and precipitates were probed with anti-Myc antibody (IB: anti-Myc). (C) Shh-N stimulates the production of Ptc-ICD7 fragments. Cells stably expressing full-length Ptc1 (with C-terminal FLAG-tag) were cultured with (+) or without (−) Shh-N-conditioned media. Solubilized cell lysates were subjected to FLAG-immunoprecipitation and subsequently probed with anti-FLAG antibody. (D) Relative luciferase activity as in (A) monitored in cells over-expressing Ptc-ICD7 fragment (pCI-Ptc1-ICD7), full-length Ptc1 (pCI-Ptc1 FL), and mock control (pCI-empty).

It was reported that Ptc1 is essential for controlling transcriptional activity of Gli1 in Shh-dependent signaling pathway [16], [17]. To examine the effects of Shh-Ptc1 pathway on the Gli1 activity in our experimental system, we performed a dual-luciferase-reporter assay with a reporter construct consisting of eight copies of a Gli-binding site (Gli-BS) present in the mouse *HNF-3β* enhancer [38], [41]. To determine the response specificity, we also used a reporter plasmid carrying a mutated version of the Gli-binding site (mGli-BS) that is unable to bind Gli1 [41]. Strong induction of luciferase was observed when the Gli-BS reporter was co-transfected with a Gli1 expression plasmid, whereas the mGli-BS reporter resulted in background levels of induction (Figure 6A). In this system, Gli1 activity was suppressed to less than 60% in cells stably expressing full-length Ptc1 in comparison with Mock-transfected HeLa cells (Figure 6A). It should be noted that the addition of a Shh-N (19 kDa form of secreted Shh-N fragment) on cells stably expressing full-length Ptc1 significantly activated the Gli1 (Figure 6A). This observation supports the notion that the stably expressed Ptc1 protein in our system is assembled properly as a functional Shh-receptor. In accordance with this view, we also confirmed that N-terminally-T7-tagged human Ptc1 in stable cells co-precipitated with Myc-tagged-Shh-N that was added in the media (Figure 6B).

Next, in an effort to determine the effect of Shh-N on the production of Ptc1 ICD7, Ptc1-stably-transfected cells were exposed to Shh-N-conditioned media. As shown in Figure 6C, exposure to the Shh-N fragment caused an increase in the production of ICD7 fragments. To address the biological activity of Ptc-ICD7 fragment, we examined the effect of Ptc-ICD7 expression on Gli1 transcriptional activity. We found that forced expression of Ptc-ICD7 fragment partly suppressed transcriptional activity of Gli1 (Figure 6D). Although the biological relevance of the Ptc-ICD7 fragment in the nucleus is still not conclusive, these observations indicate that Ptc-ICD7 fragments themselves possess some regulatory roles in the Gli1 activity.

## Discussion

In this study, we have established a cell line stably expressing full-length Ptc1 with N-terminal- and C-terminal-specific tags. Ptc1 protein thus synthesized in our stable cells could be properly assembled since Shh-N in the media was co-immunoprecipitated with Ptc1 from the solubilized extracts of these cells (Figure 6B). In addition, these cells respond to the addition of Shh-N (Figure 6A, C). With these cell lines, we found that the C-terminal cytoplasmic domain of human Ptc1 is subjected to cleavage, resulting in the generation of soluble ICD7 fragments. Importantly, we observed that the accumulation of Ptc-ICD7 fragments in the nucleus, while the N-terminal region of Ptc1 remained on the membrane fractions. Identical nuclear targeting of endogenous Ptc-ICD7 fragments was also observed with its specific antibody. These results provide the first evidence of nuclear localization of fragments derived from the intracellular domain of mammalian Ptc1. Based on these observations, we would like to propose a novel signaling pathway for mammalian Ptc1 that is mediated through the generation and nuclear translocation of ICD7 fragments.

In the classical understanding of the patched-dependent signaling pathway, the principal activity associated with Ptc1 is repression of the activity of Smo, a G-protein coupled membrane protein [5], [6], [16]–[18], [42]. The downstream target of Smo is cytoplasmic protein Suppressor of Fused (SuFu), and activation of Smo results in rapid dissociation of the SuFu-Gli complex [43]. Thus, upon binding of the Shh-ligand to Ptc1, inhibition of Smo is relieved, allowing Gli to enter the nucleus and ultimately driving expression of Hh-target genes. It has been suggested, however, that Ptc1 loss-of-function is not functionally equivalent to Smo activation in mammary epithelium, implying that Ptc1 might have Smo-independent functions in the gland [44], [45]. Indeed, it was proposed that canonical and non-canonical pathways are likely to act in parallel, as signaling that involves Hh pathway components but which is independent of Smo-mediated regulation has been reported [46]–[49]. It was also recently demonstrated that Ptc1 can stimulate ERK1/2 phosphorylation upon stimulation by Shh in the absence of Smo activity, suggesting that the Hh-signaling pathway may have acquired additional characteristics in vertebrates where direct regulation of intracellular signaling arises [49].

Although the canonical view contemplates Ptc1 protein restricted to membrane fractions, our current findings suggest that it can function as a novel signal transducers that is directly transported to the cell nucleus where it probably modulates nuclear function. The nuclear transport of cytoplasmic fragments generated from transmembrane proteins is an important mechanism of transducing signals directly from the plasma membrane to the nucleus [50], [51]. For example, Ni *et al.* reported that γ-secretase cleaved the C-terminal region of ErbB4 receptor protein, which was then translocated into the nucleus [52]. γ-secretase also cleaves the intracellular domain of Notch, generating a nuclear transcriptional regulator NICD fragment [53]. In the case of Ptc1, the C-terminal intracellular domain is similarly proteolyzed and moved into the nucleus, although our preliminary analysis indicates that γ-secretase would not be responsible for the production of C-terminal fragments (Figure S5). Identification of processing enzyme(s) as well as determination of the precise processing site(s) within Ptc-ICD7 would be necessary for future study.

Although homozygotes of *ptc1* null-knockout mice die around E9.5 with severe neural tube defects [10], an interesting viable mutant in the *ptc1* allele, named *mesenchymal dysplasia* (*mes*) has been reported [21], [54]. Ptc1 *mes* encodes a mutant Ptc1 protein lacking most of the C-terminal cytoplasmic domain [22]. *mes* homozygote mice show no gloss defects in the developing spinal cord but exhibit sterility in both sexes, and increased expression of Shh target genes (Gli1, Gli2, Ptc2 and Ptc1) in white fat tissue [55]. Importantly, the *ptc^mes^* allele is haploinsufficient over the null allele. Indeed, compound heterozygotes of *ptc-* and *mes* alleles (*ptc^−^/ptc^mes^*), which die neonatally, show a malformation of the lung, which is more severe than the viable phenotype of *mes* homozygotes [22]. Furthermore, *ptc^−^/ptc^mes^* mutant embryos showed preaxial polydactyly in all four feet with perfect penetrance. These reports also suggest that the C-terminal cytoplasmic domain of Ptc1 plays an indispensable role in mice development.

Human Ptc1 is known to functions as a tumor suppressor [3], [10]–[15], [24]–[26], [56]–[58]. Some of oncogenic mutations in *Ptc1* gene map to positions corresponding to the ICD7 region in human cancers including basal cell carcinoma (BCC) and medulloblastoma [11], [22], [24]–[26], [59]. Interestingly, it was recently reported that susceptibility to skin squamous carcinoma (SCCs) in mice is under the control of a polymorphism in the ICD7 region of Ptc1 [23]. The involvement of the Ptc-ICD7 region in the development of a variety of cancers has led to the implementation of different therapeutic strategies. Challenges for the future will be to determine how the generation of Ptc-ICD7 fragments is controlled, and what are the nuclear functions of Ptc-ICD7 fragments. Accordingly, identification of the physiological roles of nuclear Ptc-ICD7 fragments in the regulation of gene transcription and modification of tumor suppression should be future prospects.

## Materials and Methods

### Plasmid construction

The full-length cDNAs of human Ptc1 isoform L (encoding 1447 amino acids) was amplified by RT-PCR from the transcript of HEK293 cell with SuperScript™III First-Strand Synthesis System (Invitrogen, CA, USA) and KOD-FX polymerase (TOYOBO, Japan) with oligonucleotide primers as follow; sense 5′-CGACGCGTATGGCCTCGGCTGGTAACGCCG-3′, antisense 5′-GCGTCGACGTTGGAGCTGCTTCCCCGGGGC-3′. In order to express full-length Ptc1 protein with N-terminally-T7-tag and C-terminally-FLAG-tag, the PCR products were digested with *Mlu*I and *Sal*I, and inserted into the pCI-puromycin-T7-FLAG mammalian expression vectors. Note that pCI-puromycin-T7-FLAG expression vector is a derivative of pCI-neo mammalian expression vector (Promega, WI, USA) and contains CMV enhancer/promoter. pCI-puromycin-T7-FLAG vector was design to include three repeats of a T7-tag at N-terminus and three repeats of FLAG-tag at C-terminus of its protein product. Similarly, we also prepare an expression vector for human full-length Ptc1 with N-terminally-3×T7-tag and C-terminally-2×S-tag. A cDNA encoding the Ptc- ICD7 fragment (C-terminal cytoplasmic domain consisting of residues 1162–1447 of human Ptc1) was PCR amplified with KOD-FX polymerase and was ligated to pCI-neo mammalian expression vector (Promega) with C-terminal FLAG-tag. For expression of nematode TRA-2 ICD7 (amino acids 1135 to 1475 of *C. elegans* TRA-2 protein) [33], corresponding cDNA was subcloned into pCI-neo expression vector with N-terminal FLAG-tag. Human Shh cDNA was amplified by RT-PCR from the transcript of HEK293 cell and the cDNA fragment was subcloned into pCI-neo-6×Myc-2×S expression vector. This vector was design to include six repeats of Myc-tag at N-terminus and two repeats of S-tag at C-terminus of full-length Shh protein. Sequences of all plasmids were verified before transfection experiments.

### Mammalian cell culture and transfection

HeLa cell [60] was cultured in Dulbecco's modified Eagle's medium (Sigma Chemical Co., St Louis, MO) supplemented with 10% heat-inactivated Calf Serum at 37°C under a 5% CO_2_ atmosphere. Mouse C3H10T1/2 cells and embryonic primary cells were cultured as is HeLa cells except for the usage of 10% heat-inactivated Fetal Calf Serum.

Transfection of the expression vectors to HeLa cells were performed with HilyMax transfection reagent (Dojindo, Japan) according to the protocol supplied by the manufacturer. The total amount of plasmid DNA was adjusted to 1 µg with an empty vector. At 24 hr after transfection, the cells were harvested and subjected to immunological analysis unless otherwise noted.

For cycloheximide treatment, pCI-neo expression vector that encoding C-terminally-FLAG-tagged Ptc-ICD7 was transfected into HeLa cells. At the time of cDNA transfection, protein synthesis inhibitor cyclohexamide was added with various concentrations. We found that 12.5 µg/mL cyclohexamide treatment completely suppressed protein expression from transfected expression vector. Cells were harvested 24 hr after transfections for immunological analysis.

To establish stable cell lines that express a moderate level of human full-length Ptc1 protein, HeLa cells were transfected with pCI-puromycin-3×T7-Ptc1-3×FLAG mammalian expression vectors and cultured for one week in the presence of 1.5 µg/mL puromycin, and colonies that survived were picked up into 24-well multidish. The cells were culture for another two weeks, and stable expression of exogenous Ptc1 protein was verified with anti-FLAG-tag and anti-T7-tag antibodies.

To prepare a Shh-N-conditioned media, an expression vector that encodes full-length N-terminally-Myc-tagged and C-terminally-S-tagged Shh was transfected into HeLa or HEK293 cells. At 24 hr after Shh transfection, we confirmed the appearance of the matured form of Myc-tagged Shh (with a molecular weight of 19 kDa) in the culture media of both HeLa and HEL293 cells. To remove the non-processed form of Shh (45 kDa form with a C-terminal S-tag, possibly contaminated from damaged cells), we pre-cleared the medium with S-agarose beads. To examine the effect of Shh-N, cells stably expressing human full-length Ptc1 with an N-terminal T7-tag and a C-terminal FLAG-tag were exposed to Shh-N-conditioned media for 4 hr.

### Immunological analysis

The anti-Ptc1 ICD7 C-terminal antibody (designated anti-Ptc-ICD7 (1420–1434) antibody) was prepared for this study as follows. The antigenic peptide used in this study corresponds to human Ptc1 C-terminal amino acids sequence 1420–1434 (CERRDSKVEVIELQD) that is a region specific to Ptc1 among the predicted human protein sequences from the genome database. Immunization to the rabbit with 0.3 mg antigenic peptide was performed for six times at two-week intervals by Operon Biotechnologies (Tokyo, Japan). The antibody was successively purified with Protein A-Sepharose and antigenic peptide affinity chromatography. Antigenic sequence is completely conserved between human and mouse, and we confirmed that the antibody thus obtained can efficiently recognize both of human and mouse Ptc1.

For immunoprecipitation analysis, cells were washed with ice-cold phosphate-buffered saline and lysed with immunoprecipitation (IP) buffer containing 20 mM Tris-HCl, pH 7.5, 150 mM NaCl, 5 mM EDTA, 1% NP-40, 10 mM N-ethylmaleimide, 100 µM MG132 and Complete™ protease inhibitor cocktail (GE healthcare). The lysate was sonicated for one second and centrifuged at 20,000× g for 10 min at 4°C, and the resulting supernatant was incubated with 3 µL of anti-Flag M2-agarose beads (Sigma) for 2 hr at 4°C. After the beads had been washed five times with the IP buffer, the precipitated immunocomplexes were subjected to SDS-PAGE.

For Western blot analysis, the whole cell lysate and immunoprecipitates were separated by SDS-PAGE and transferred onto PVDF membranes (GE Healthcare). The membranes were probed with specific antibodies as indicated and then incubated with horseradish peroxidase-conjugated antibody against mouse and rabbit immunoglobulin (GE Healthcare), followed by detection with ECL Western blotting detection reagents (GE healthcare).

The following antibodies were used for immunological analyses in this study: anti-FLAG polyclonal (Sigma), anti-FLAG M2 monoclonal (Sigma), anti-T7 tag^R^ monoclonal (Novagen), anti-Ptc-ICD7 C-terminal (1420–1434) (prepared in this study), anti-human Ptc1 SSD (500–600) (Abcam, Cat. No. ab39266), anti-human Ptc1 ICD7 peptide (_1271-_HPESRHHPPSNPRQQ_-1285_) antibody (Abcam, Cat. No. 51983), anti-TRA-2 ICD7 [33], anti-tubulin (ICN), anti-BIP (BD Bioscience), anti-Histone H2B (Santa Cruz), Alexa Fluor^R^488 anti-rabbit IgG (Invitrogen), Alexa Fluor^R^488 anti-mouse IgG (Invitrogen), Alexa Fluor^R^568 anti-mouse IgG (Invitrogen), HRP-conjugated anti-rabbit IgG (GE healthcare), and HRP-conjugated anti-mouse IgG (GE healthcare).

### Microscopic observations

For immunocytochemical observations of cultured cells, cells were grown on micro coverglass (Matsunami, Japan), fixed by incubating in 4% paraformaldehyde, and were then permeabilized with 0.1% Triton X-100. Fixed cells were blocked with 3% calf serum in PBS and reacted with a series of primary antibodies at room temperature for one hour. For examples, affinity-purified anti-Ptc-ICD7 C-terminal (1420–1434) antibody was diluted to a concentration of 5.5 nM (about 0.8 µg/mL) before incubation. For peptide competition experiments, an antigenic peptide was used at concentrations of 1.2 µM or 0.6 µM. Anti-T7-tag and anti-FLAG-tag antibodies were used at 15 nM. Alexa™ 488-conjugated anti-rabbit IgG antibody and Alexa™ 594-conjugated anti-mouse IgG antibody (Molecular Probes) were used as secondary antibodies at 1∶800 dilution. To observe the nucleus, cells were treated with 2.5 µg/ml Hoechst 33342 or DAPI for 15 minutes at 25°C and then washed another three times in PBS-T. Immunofluorescent images were obtained with BIOREVO BZ9000 fluorescence microscope (Keyence, Japan) and an LSM510 invert confocal microscopy system (Carl Zeiss, Germany).

### Subcellular fractionation

Subcellular fractionation of HeLa cells was performed as follows. (Step I) stably transfected cells were harvested and suspended in 200 µL of Solution buffer (20 mM HEPES, pH 7.4, 1 mM EDTA and 0.25 M sucrose) by 15 strokes of a 27G needle. After centrifugation at 1,000×g for 10 min, the supernatants (post-nuclear fraction) were collected as cytoplasmic fractions. (Step II) the pellets were re-suspended in 400 µL of Fraction buffer (20 mM HEPES, pH 7.4, 3 mM MgCl_2_ and 10 mM NaCl), incubated for 10 min on ice, added NP-40 to final concentration of 0.5% and centrifugation at 10,000×g for 20 sec after voltex mixing. Step II was repeated twice and resulting pellets were collected as nuclear fractions.

For C3H10T1/2 cell fractionation, cells were suspended in 200 µL of Low Osmotic buffer (10 mM HEPES, pH 7.9, 10 mM KCl, 1.5 mM MgCl_2_, 10 mM DTT and 1.5% NP-40) with pipetting 10 times. After centrifugation at 750×g for 10 min, the supernatants (post-nuclear fraction) were collected as the cytoplasmic fraction. The pellets were re-suspended in 500 µL of Low Osmotic buffer with pipetting 10 times. After centrifugation at 750×g for 10 min, the pellets were suspended in 500 µL of cold PBS and centrifuged at 750×g for 10 min. After repeating PBS re-suspension twice, resulting pellets were collected as the nuclear fraction. The nuclear fraction was sonicated with SDS sample buffer and subjected to electrophoresis analysis with the cytoplasmic fraction.

### Luciferase assay

The reporter plasmids 8×3′Gli-BSδ51LucII and 8×m3′Gli-BSδ51LucII were constructed by placing eight copies of a Gli-binding site element present in the mouse *HNF-3β* enhancer or a mutated version of it [41]. After cDNA transfection, HeLa cells were harvested and transcriptional activity of Gli1 was monitored by the Dual-Luciferase^R^ Reporter Assay System (Promega) according to the protocol supplied by the manufacturer. Normalization of transfection efficiencies was carried out using *Renilla* luciferase activities. All reporter assay experiments were repeated at least three times, and transfection was done in triplicate. Reporter plasmids 8×3′Gli-BSδ51LucII and 8×m3′Gli-BSδ51LucII as well as Gli1 expression vector (pcDNA3.1-HisB-Gli1) were generous gifts from Dr. H. Sasaki (Kumamoto Univ., Japan).

## Supporting Information

Figure S1 **Comparison of nematode TRA-2 and human Ptc1.** (A, B) Hydropathy analyses of human Ptc1 and nematode TRA-2 sequences suggest that both proteins are composed of 12 membrane-spanning helices like members of transporter superfamily. Both Ptc1 and TRA-2 proteins appear to be topologically duplicated as it is composed of tandemly-repeated motif of six hydrophobic domains. Ptc1 and TRA-2 share similar structure, implying that they originate from common ancestor. (C) Amino acid sequence alignment of seventh intracellular domain of human Ptc1 (Ptc-ICD7) and *C. elegans* TRA-2 (TRA-2 ICD7). Identical amino acids are boxed by red, and related amino acids are boxed by orange. Antigenic sequence of anti-Ptc-ICD7 (1420–1434) antibody was indicated with underline. Amino acid number was derived from human Ptc1 isoform L.
(EPS)

Figure S2 **TRA-2 ICD7 is localized in the nucleus.** (A) Nuclear localization of endogenous TRA-2 ICD7 fragment in wild-type young adult hermaphrodite nematodes. Nuclei of intestinal cells were specifically stained with an anti-TRA-2 ICD7 antibody, while the nuclei were scarcely stained with preimmune IgG. Note that each photograph were taken with identical exposure time. Immuno-staining in green, Hoechst DNA stain in blue. Scale bar, 100 µm. (B) Flag-tagged TRA-2 ICD7 protein (fragments of amino acids 1135–1475 of TRA-2) was subcloned into pCI-neo expression vector and the protein fragment was transiently expressed in HeLa cells. Forced expression of TRA-2 ICD7 fragment results in accumulation in the nucleus of HeLa cells. TRA-2 ICD7-positive nuclei were indicated by arrowheads. Scale bar, 20 µm.
(EPS)

Figure S3 **Comparison of expression level of Ptc1 in HeLa cells.** Ectopically-expressed Ptc1 proteins were probed with anti-Ptc-ICD7 antibody. Note that production of full-length Ptc1 by “transient” expression results in much larger amounts of accumulation compared to that of “stable” expression.
(EPS)

Figure S4 **Ectopically-expressed Ptc1 protein detected with anti-Ptc-ICD7 (1420–1434) antibody in C3H10T1/2 cells.** Human full-length Ptc1 cDNA in an expression vector was transiently transfected into C3H10T1/2 cells, and whole cell lysates were immunoblotted with anti-Ptc-ICD7 (1420–1434) antibody. Mock transfected cells were used as a negative control.
(EPS)

Figure S5 **Effects of protease inhibitors on the appearance of Ptc1 C-terminal fragments.** HeLa cells stably expressing full-length Ptc1 were treated with a series of protease inhibitors at 20 µM for 13 hr. C-terminal ICD7 fragments were detected by anti-FLAG antibody, while the full-length form of Ptc1 (Ptc1 FL) was detected with N-terminal T7-tag. Anti-actin immunoblots were used as loading control.
(EPS)
